# Supplementary material for: WordCommentsAnalyzer: A windows software tool for qualitative research
Source: F1000Res. 2018 Sep 4;7:536. Originally published 2018 May 3. [Version 2] doi: 10.12688/f1000research.14819.2 (PMC6137411; doi:10.12688/f1000research.14819.2)
Supplement: Supplementary file 3 [file f1000research-7-17614-s0002.tgz › b2a79496-4ada-47c4-9682-a99ce867836d.zip!/Eight Iranian Health Journals/Yafteh _3.docx]

© Yafteh  . This work is licensed under CC BY 4.0 (https://creativecommons.org/licenses/by/4.0/).

Volume & Issue: دوره 19 - شماره 3

Title: بیو تروریسم در آب و مواد غذایی

http://yafte.lums.ac.ir/article-1-2566-fa.html

مقدمه: بیوتروریسم بمعنای رهاسازی عمدی میکروارگانیسمهای شدیدا بیماری زا و یا سموم آنهاست که باعث مرگ انسانها، گیاهان وحیوانات میشود. مواد و روش ­ها: این مقاله به شیوه مروری و با استفاده از منابع کتابخانه ای تدوین شده است. یافته ­ها: یکی از راه های انتشار عوامل بیولوژیک دربین جمعیت هدف، آلودگی عمدی آب و مواد غذایی است. آب و مواد غذایی آلوده شده در فعالیتهای بیوتروریستی، توسط ویژگیهای ارگانولپتیک قابل تشخیص نبوده و در اکثر مواقع خاموش،  ناگهانی و بدون تغییرات ظاهری (رنگ،  بو،  طعم) بروز می نمایند. تصفیه خانه ها، چاه های مورد استفاده برای تأمین آب شرب، مخازن آب تصفیه شده، انبارهای نگهداری و مراحل مختلف تولید،  نگهداری و توزیع مواد غذایی درکارخانجات مواد غذایی و مواد غذایی وارداتی، بهترین نقاط برای وارد نمودن این عوامل بوده و هر چه سیستم ها و مکانیسم های کنترلی ابتدایی تر، غیر صنعتی تر و غیر بهداشتی تر باشند امکان نفوذ و خرابکاری در آنها بیشتر است. با توجه به مقاومت زیاد، عدم تشخیص سریع، پایین بودن دوزعفونی و عدم وجود واکسن در بسیاری از عوامل بهترین راه مبارزه پیشگیری می­باشد. بحث و نتیجه ­گیری: با توجه به اهمیت این مسئله و صدمات جبران ناپذیری آن و دشمنان بسیار نظام مقدس جمهوری اسلامی ایران در این مقاله به توصیف این پدیده و راههای کنترل و پیشگیری آن پرداخته شده است.

Title: بیماری ویروسی ابولا: یک تهدید جدی

http://yafte.lums.ac.ir/article-1-2567-fa.html

مقدمه: بیماری ویروسی ابولا (EVD) به عنوان تب خونریزی دهنده، بیماری شدید و اغلب کشنده در بین انسان ها شناخته شده است. این ویروس از طریق تماس با خون یا مایعات بدن فرد مبتلا، اشیاء آلوده مانند سوزن، حیوانات آلوده یا گوشت حیوانات وحشی انتقال می یابد. تاریخچه میزان مرگ و میر این عفونت بیشتر از 80  درصد بوده اما اقدامات مدرن پزشکی و بهداشت عمومی تا حدی قادر به کاهش این رقم و تأثیر ابولا بر افراد و جوامع بوده است. در حال حاضر هیچ درمان استانداردی برای ابولا وجود ندارد، لذا پیشگیری از عفونت و جلوگیری از گسترش بیشتر ویروس مهم است. با توجه به این که میزان مرگ و میر ناشی از این عفونت بالا بوده و درمان اختصاصی برای آن وجود ندارد و همچنین امکان استفاده از آن به عنوان سلاح بیولوژیک در جنگ ها مطرح می باشد لذا مروری بر اپیدمیولوژی، علائم بالینی و آزمایشگاهی و مدیریت این بیماری به عنوان اولین گام جهت تشخیص صحیح و سریع و عکس العمل به موقع ضرورت دارد. در این راستا مقاله مروری حاضر با استفاده از کلید واژه­های بیماری ویروسی ابولا، اپیدمیولوژی، تشخیص، درمان، پیشگیری و بیوتروریسم در پایگاه­های داده­ای Pubmed، Elsevier، Google Scholar، SID، Google و سایر وب سایت ها انجام شد.

Title: اگروتروریسم، تعاریف، اثرات و راهکارهای پیشگیری

http://yafte.lums.ac.ir/article-1-2571-fa.html

مقدمه: امنیت غذایی به معنی دسترسی همه مردم در تمام اوقات به غذای کافی برای داشتن یک زندگی سالم است که در سه جنبه موجود بودن غذا، دسترسی به غذا و پایداری در دریافت غذا قابل بررسی است. از طرفی، اگروتروریسم زیر مجموعه بیوتروریسم است که در آن، فرد یا گروهی از افراد با وارد نمودن عمدی انواعی از باکتریها، ویروس ها و یا قارچها به زمین های کشاورزی و یا محل پرورش و نگهداری دام و طیور باعث آلودگی و بیماری دام و طیور و محصولات کشاورزی می گردند. هدف از این مقاله بررسی خطراتی چون اگروتروریسم، راهکارهایی برای پیشگیری از ارگوتروریسم  و مدیریت امنیت غذایی و بهبود آن در کشور است. مواد و روش‌ ها: در این مطالعه مروری، مقالات پژوهشی معتبر در مورد اگروتروریسم، بدون محدوده زمانی و زبان انتشار و با استفاده از واژگان کلیدی " اگروتروریسم، بیوتروریسم، امنیت غذائی و ناامنی غذائی، پیشگیری، اثرات " در پایگاه های اطلاعاتیPubMed, Elsevier ,SID, Medlib, Magiran, IranMedex, IranDoc, Google Scholar, Scopus  جستجو شد. در نهایت 20 مقاله کاملاً مرتبط مورد بررسی قرار گرفت. یافته ­ها: نتایج نشان داد که حملات تروریستی علیه کشاورزی تازگی ندارد و در طی تاریخ بوسیله دولتها یا از طریق سازمانهای تروریستی مستقل و تابع دولتهای خاص، انجام شده است.  همچنین، اثرات تخریبی ناشی از اگروتروریسم در اقتصاد و امنیت ملی و فراملی کشورها، به مراتب از اثرات مخرب ناشی از جنگ افزارهای متعارف که در اختیار سازمانهای نظامی و شبهه نظامی قرار دارد، بیشتر می­باشد. بطوریکه می‌تواند منجر به از بین رفتن  زنجیره تولید ﻏﺬایﺟﺎﻣﻌﻪ، خسارت‌های اﻗﺘﺼﺎدی، ﻧﺎﺑﻮدی ﺻﻨﻌﺖ ﮐﺸﺎورزی یﮏ ﮐﺸﻮر، ایﺠﺎد ﻫﺮج و ﻣﺮج و در ‌ﻧﻬﺎیﺖ از ﺑﯿﻦ ﺑﺮدن ﺗﻮان ﻣﻘﺎوﻣﺖ ﮐﺸﻮر و در ﻧﺘﯿﺠﻪ ﺷﮑﺴﺖ ﮐﺎﻣﻞ ﮐﺸﻮر ﻫﺪف ﺑﺎ ﮐﻤﺘﺮیﻦ ﻫﺰیﻨﻪ شود. نتیجه گیری: هدف از حملات اگروتروریستی ایجاد ترس و وحشت در بین مردم، کاهش دسترسی مردم به مواد غذائی سالم و در نتیجه ایجاد نا امنی غذائی در جامعه، بی اعتمادی مردم نسبت به دولتها و در نهایت صدمه زدن به ساختار سیاسی حکومتها و حتی براندازی یک حکومت یا دولت  است.

Title: بیوتروریسم، تهدیدی برای سلامت عمومی

http://yafte.lums.ac.ir/article-1-2573-fa.html

مقدمه: امروزه، حملات دشمن در استفاده از ابزار نظامی خلاصه نمی شود. تجربه­های ناموفق نظامی باعث گرایش کشورهای متخاصم به شیوه­ های نوین شده است. استفاده از باکتری ها، ویروس ­ها و سموم را که باعث آسیب به سلامت افراد می شود، بیوتروریسم می گویند. آگاهی نسبت به بیوتروریسم را باید در زمره اهداف پدافند غیرعامل دانست. مواد و روش‌ها: برای آشنایی با تاریخچه و چگونگی بیوتروریسم، این مطالعه انجام شد. با استفاده از کلید واژه­های لاتین بیوتروریسم، عوامل بیولوژیک، سلامت عمومی، پدافند غیرعامل و کلمات معادل فارسی، به مطالعه مراجع مرتبط (بخصوص در متون پزشکی) خارجی و داخلی پرداخته شد. بعد از استخراج اطلاعات اولیه، تحلیل نهایی با دانش اولیه محققین صورت گرفت. یافته ­ها: قدمت بیوتروریسم به قبل از میلاد مسیح بر می­ گردد. پیشرفت دانش بشر، امروزه باعث استفاده از داروها و عوامل میکروبی و دستکاری­ های ژنتیکی شده است. از شایعترین عوامل میکروبی به عامل بیماری سیاه زخم، طاعون، تولارمی، بوتولیسم، تب­های خونریزی دهنده، ویروس­ های تنفسی میتوان اشاره کرد. شش علامت شایع این بیماری­ ها شامل: اختلالات تنفسی، تب و خونریزی، تب و ضایعه پوستی، مننژیت و انسفالیت، فلج اندام و انواع اسهال ­ها می­ باشد. نتیجه گیری: به علت تابلوهای بالینی متفاوت تیم ­های بهداشتی و درمانی باید خود را آماده مقابله با هر نوع تظاهر غیرعادی در حملات بیوتروریسم کنند. ارجاع سریع موارد مشکوک و اطلاع رسانی به مسئولین بهداشتی ضروری است. این راهکارها می تواند به نجات مصدومین منتهی شود.

Title: راههای تامین امنیت غذایی و جایگاه آن در پدافند غیر عامل

http://yafte.lums.ac.ir/article-1-2578-fa.html

مقدمه: با توجه به نقش نیروی انسانی سالم در توسعه یک کشور و اهمیت تغذیه و غذای سالم در سلامت انسان بعنوان محور توسعه کشور و مقابله با تهدید های خارجی در این مقاله ابتدا به تعریف امنیت غذایی ، وضعیت امنیت غذایی در ایران ونقش آن در مقابله با تهدیدات پرداخته می شود و سپس  راهکارهای موجود و استفاده از پتانسیل بومی برای ارتقاء امنیت غذایی مطرح می گردد. مواد و روش‌ها: در این مطالعه از تجربیات نویسنده و سوابق و متون علمی مرتبط برای واکاوی موضوع و تحلیل آن استفاده شده است. یافته ­ها: برای جلوگیری از تهدیدات خارجی در زمینه داشتن غذای سالم و ایمن و در عین حال مغذی نیازمند سیستمی هستیم که به خودمان تکیه داشته باشد و تهدیدات خارجی نتواند بر این دسترسی تاثیرگذار باشد. در کشور ما شرایط اقلیمی متفاوت، تنوع گونه های گیاهی خوراکی وحشی در طبیعت ایران و وجود امکانات و شرایط در استانهای مختلف برای تولید غذای سالم و محلی پتانسیل خوبی را برای مقابله با ناامنی غذایی و داشت  جامع ه­ای سالم فراهم نموده است. نتیجه گیری: با توجه به اینکه در امنیت غذایی، تهیه غذای سالم و مغذی و دسترسی افراد از نظر اقتصادی، اجتماعی در همه زمان ها مدنظر است نتایج مطالعه حاضر نشان داد که در حال حاضر ناامنی غذایی در کشور بالاست و تهدید کننده می باشد لذا برای تامین امنیت غذایی و افزایش دسترسی به غذای سالم و مغذی استفاده از پتانسیل مواد غذایی بومی و محلی از جمله مواد غذایی کشت شده و خودرو محلی توصیه می شود.

Title: بررسی تجویز و مصرف آنتی بیوتیک و نقش آن در مقاومت میکروبی و اثرات آن بر اقتصاد مقاومتی

http://yafte.lums.ac.ir/article-1-2591-fa.html

مقدمه: مصرف ‏بیرویه آنتی بیوتیک‌ها، باعث مقاومت میکروبی شده بنحوی که مصرف خودسرانه آنها باعث بالاتر رفتن مقاومت میکروبی و نهایتا ًسبب گردیده ما نیاز به آنتی بیوتیک‌های جدید و قوی تر داشته باشیم که مشکل این نوع آنتی بیوتیک‌های جدید"هزینه بسیار ‏بالا و کمرشکن" آنهاست. ‏امروزه در جامعه مواردی نظیر آنتی بیوتیکها و غذاهای وارداتی و تأثیر آن بر سلامت و مصرف بی‌رویه آنتی‌بیوتیک و ضررهای آن مغفول مانده است. مشکل موجود در این حوزه اجرایی نشدن یا فقدان دستورالعمل‌های لازم است لذا در این مقاله بر آن شدیم  به بررسی موضوع و راه حل دهیم. مواد و روش‌ها: در این مطالعه بیش از یکصد سایت و مقالات مرتبط که یا خود یا دانشمندان دیگر مستند شده و مجموعه تجربیات و مقالاتی که در طی بیست سال اخیر در زمینه بیماریهای عفونی و میکروبی  بدست آورده ایم و همچنین مقالات نمایه شده ISI، DOAJ، SCOPUS و غیره استفاده شده است. نتیجه گیری: بصورت درمان بیماران عفونی با آنتی بیوتیکهای ایرانی خودکفایی در تولید واکسن، تقویت روشهای تشخیص دقیق عفونتها تحقیقات کاربردی و عملیاتی و هدفمند نمودن مصرف و تجویز منطقی آنتی بیوتیکها جهت رسیدن به اقتصاد مقاومتی می باشد که به تفصیل در متن به آن خواهیم پرداخت.

Title: تبیین شاخص های شخصیت طراز انقلاب اسلامی در راستای تقویت نفوذ ناپذیری (از منظر نهج البلاغه)

http://yafte.lums.ac.ir/article-1-2582-fa.html

تمام انقلابهایی که در طول تاریخ تحقق پیدا کرده اند دو مرحله سخت را پشت سر گذاشته اند که از اهمیت ویژه ای برخودار می باشد، یکی وقوع اصل انقلاب و دیگری ثبات و استمرار انقلاب در راستای رسیدن به اهداف، می باشد، در بین این دو اصل آنچه دشوارتر و پیچیده تر است حفظ و نگهداری انقلاب است، انقلاب اسلامی ایران مانند دیگر انقلابها از این امر مستثنی نبوده و باید در جهت استمرار و ثبات و تحقق آرمانهایش با جدیت از همه ظرفیتش استفاده کند. از این رو در این نوشتار سعی شده با توجه به عامل اصلی و پیش برنده انقلاب یعنی مردمِ انقلابی، شاخص های مهم افراد طراز انقلاب را در راستای اصل نفوذ ناپذیری، بیان کرده و با تبیین این شاخص ها فرد طراز انقلاب را معرفی کنیم، شخصیتی که باید هم شاخص تدین و هم شاخص اثرگذاری به عنوان خواص جامعه و هم شاخص روحیه ی انقلابی گری و بصیرت را در خود جمع کند و از عوامل و زمینه های برون رفت از دایره شخصیت طراز انقلاب، پرهیز کند. لذا می کوشیم که در قالب معرفی شخصیت طراز انقلاب، به نوعی فرصت ها و تهدیدهای موجود در عرصه جامعه اسلامی را شناخته و معرفی کنیم، خطراتی که گاها ممکن است از جنس و دل همین مردم به ظاهر انقلابی ظهور و بروز می کند، از آنجایی که حکومت زمان حضرت علی علیه السلام درسها و عبرتهایی در خور تامل و نزدیک به انقلاب ما دارد، در سددیم که  از منظر حضرت علی علیه السلام در نهج البلاغه، به تبیین نشانه ها و علائمی که باید یک شخصیت انقلابی داشته باشد، پرداخته و عیار و طرازی که ترسیم کننده این شخصیت است مطرح شود، چرا که عده ای هر چند ممکن است هم دیندار بوده و هم از خواص باشند اما انقلابی نباشند.

Title: فیزیوپاتولوژی انحراف نرم جامعه ی اسلامی در نهج البلاغه؛ با تأکید بر نقش خواص گمراه

http://yafte.lums.ac.ir/article-1-2583-fa.html

مقدمه: اصطلاح جنگ نرم، گرچه نوظهور است اما در عمل از قدیم، از طرف دشمنان اسلام، در جامعه اسلامی،پیاده شده است. بنابراین رگه­های آسیب شناسی جنگ نرم در متون دینی قابل ردگیری است. حضرت علی (ع) به دلیل حکومت کوتاه و پر برکت خویش، بیش از سایر اهل بیت، در نهج ­البلاغه، به این مهم پرداخته اند. نقش خواص گمراه در جنگ نرم قابل توجه است. منظور از خواص در این نوشتار، کسانی است که به هر دلیل-حق یا ناحق- مورد توجه و اقتدای دیگران هستند. مواد و روش‌ها: مقاله کنونی با روش کتابخانه ای و تحلیل محتوا به بررسی نقش خواص گمراه در جنگ نرم و تبدیل تدریجی و نا محسوس ضدارزش ها به ارزش و قداست زدایی نظری و عملی از ارزش ها در جامعه اسلامی می پردازد. یافته ­ها: انحراف جامعه اسلامی، از اندیشه یا رفتار خواص آغاز می شود. در ادامه، انحراف به صورت "شبهه در اندیشه" و "شهوت در رفتار" به توده­ی مردم سرایت می کند. ابتدا، به دلیل تعارض عقاید اسلامی با رفتار هوسناک جامعه، عذاب وجدان جمعی شکل می گیرد. در اینجا خیانت مجدد خواص، به صورت تئوریزه کردن هوسها و ضد ارزشها در قالب شبهه و شبهوت بروز می کند. نتیجه گیری: در نهایت، عذاب وجدان جمعی، جای خود را به یک آرامش کاذب روانی می­دهد و جامعه به "سرگردانی در اندیشه" و "مستی در عمل" مبتلا  و از فهم حق ناتوان می شود. اصلاح چنان جامعه ای دشوار است.
